# Imperfect Breathing

**Published:** 1878-05

**Authors:** 


IMPERFECT BREATHING. IMPURE BLOOD.

Surely it will not be difficult to remember these two things. We thus can plainly see how it is that persons who sit a great deal become consumptive; and any one may apply it to himself in the various occupations of life, without any further specifications as to this branch of the causes of Consumption.

More time will be spent in considering the other great branch of causes, *Impure Blood*, because it is not generally understood what are the more general causes of impure blood, and they ought to be generally known.

The heart has two suits of rooms, one filled with impure blood, going to the lungs to be purified; the other containing the purest blood of the body, which having undergone purification and perfection in the lungs, has been returned to this other side of the heart, to be propelled therefrom to the most distant portions of the human frame, imparting in its progress, renovation, restoration and life. The right side of the heart contains the impure, imperfect blood, while the pure blood is found in the left. But it cannot get from the right side into the left, without passing through an out-house, the Lungs, where the purifying process is carried on; and how?

We have seen that the blood is in the little branches of blood-vessels spread like a vine on the walls of the air-cells, the lungs, distended by air. Now, the blood does not come in actual contact with the air, the membrane of these minute vessels, thinner than the thinnest paper, manufactured only in Heaven, by omnipotent skill for the express purpose, is between the air and the blood. But a most wonderful process goes on here; there is a passage of substances through these membranes, the life of the air, the oxygen, as we say, passes out of the air-cell into the blood in the blood-vessels, and the impurities, the death of the blood passes from the blood-vessel into the air-cell, and in a moment the dead blood is made alive, and the air so pure from without but a moment before, is now deadly. Sc the death of the blood and the life of the air pass through these membranes, as light passes through glass or as electricity along the wires. Thus the Lungs are the great 'Change of life —the market place where Vitality and Death change their wares, the air being the nobler of the two, for while it takes death from the blood, it gives its own life therefor, the savior of physical humanity.

Let the most careless reader note and feel here, how impossible it is for the blood to be purified unless he breathes abundant pure air. The importance of breathing it constantly is strikingly exhibited in the established fact, that every ounce of blood of the whole body is thus aired every two and a half minutes of our existence. Thus the breathing of a pure air for so short a time as two and a half minutes imparts purification and refreshment to the whole human frame. This explains the instantaneousness with which persons are revived when taken into the air after confinement to a close room or crowded apartment for some time.

